# A lightweight hybrid deep learning system for cardiac valvular disease classification

**DOI:** 10.1038/s41598-022-18293-7

**Published:** 2022-08-22

**Authors:** Yazan Al-Issa, Ali Mohammad Alqudah

**Affiliations:** 1 Department of Computer Engineering, Yarmouk University, Irbid, 21163 Jordan; 2 Department of Biomedical Systems and Informatics Engineering, Yarmouk University, Irbid, 21163 Jordan

**Keywords:** Health care, Engineering

## Abstract

Cardiovascular diseases (CVDs) are a prominent cause of death globally. The introduction of medical big data and Artificial Intelligence (AI) technology encouraged the effort to develop and deploy deep learning models for distinguishing heart sound abnormalities. These systems employ phonocardiogram (PCG) signals because of their lack of sophistication and cost-effectiveness. Automated and early diagnosis of cardiovascular diseases (CVDs) helps alleviate deadly complications. In this research, a cardiac diagnostic system that combined CNN and LSTM components was developed, it uses phonocardiogram (PCG) signals, and utilizes either augmented or non-augmented datasets. The proposed model discriminates five heart valvular conditions, namely normal, Aortic Stenosis (AS), Mitral Regurgitation (MR), Mitral Stenosis (MS), and Mitral Valve Prolapse (MVP). The findings demonstrate that the suggested end-to-end architecture yields outstanding performance concerning all important evaluation metrics. For the five classes problem using the open heart sound dataset, accuracy was 98.5%, F1-score was 98.501%, and Area Under the Curve (AUC) was 0.9978 for the non-augmented dataset and accuracy was 99.87%, F1-score was 99.87%, and AUC was 0.9985 for the augmented dataset. Model performance was further evaluated using the PhysioNet/Computing in Cardiology 2016 challenge dataset, for the two classes problem, accuracy was 93.76%, F1-score was 85.59%, and AUC was 0.9505. The achieved results show that the proposed system outperforms all previous works that use the same audio signal databases. In the future, the findings will help build a multimodal structure that uses both PCG and ECG signals.

## Introduction

Cardiovascular diseases (CVD) are a leading cause of death and they claimed the lives of 18 million people in 2015 worldwide^1,2^. They are caused primarily by high blood pressure, tobacco, diabetes, lack of exercise, and obesity^1^. The heart is a vital body organ, a mechanical device, and any abnormality is reflected in the heart sound and propagates through the chest wall. A lot of information about overall heart health can be obtained using conventional methods that collect different heart sounds using a stethoscope. The problem with classical methods is that they are subjective and various physicians might have different interpretations. Needless to say, misdiagnosing heart irregularities can be fatal since remedying risk factors can prevent 90% of heart disorders^3^.

As a result, early and quick detection of heart problems is critical in eliminating serious complications. The heart is made up of four chambers, there are four heart valves, the aortic, and the mitral on the left heart, and the pulmonary and tricuspid on the right heart. The main function of the valves is to regulate the blood flow in the circulatory system. Heart Valvular Disease (HVD) is a type of cardiovascular disease that results from the blocking, hardening, or malfunctioning of the heart valves and this can be caused by aging, dysplasia, calcific disease, inflammatory disorders, and connective tissue disorders^4^.

The main consequence of a heart valve defect is “stenosis” described as a narrowing of the heart valve preventing blood discharge. Another consequence is “regurgitation” illustrated by the inability of the valve to prevent blood backflow^5^. The common signs of a damaged heart valve are fatigue, palpitations, shortness of breath, weakness, fainting, and chest pain^6^. Recognizing the warning signs early can help prevent a heart attack or stroke.

A healthy human heart generates a unique murmur, any irregularity is reflected in this sound and can be easily picked up using a stethoscope. Over the years much effort was spent to construct an automatic cardiac diagnostic system that employs machine and deep learning techniques and uses Phonocardiograms (PCGs)^7^. Besides heart sounds different other modalities like Electrocardiograms (ECG), Computed Tomography (CT), and Magnetic Resonance Imaging (MRI) are being used for the effective screening of heart abnormalities. The widespread use of auscultation is a result of it being a simple, cost-effective, non-invasive, and reliable tool for detecting heart anomalies. Phonocardiograms (PCGs) can help physicians visualize the waveform generated by the heart and can use it to manually extract features that are correlated with different heart conditions. Only a handful of scholars tried to solve the two-class (normal vs. abnormal) problem^8–10^, while numerous researchers attempted to solve the five-class problems^11–20^.

The purpose of this study is to investigate the efficiency of using Deep Learning (DL) techniques, particularly Convolutional Neural Networks (CNN) to discriminate between different heart valvular anomalies. A light cardiac diagnostic system trained using the original unfiltered phonocardiograms (PCG) obtained from publicly available datasets^12,13^ is built. The hybrid model is proposed that combines the use of Convolutional Neural Network (CNN) and Long Short-Term Memory (LSTM). The CNN is used for automatic feature extraction whereas the LSTM is used for classification purposes. The study distinguishes normal and abnormal instances (binary), it also discriminates between five categories (multiclass) of heart valvular conditions, namely normal, Aortic Stenosis (AS), Mitral Regurgitation (MR), Mitral Stenosis (MS), and Mitral Valve Prolapse (MVP) in a non-invasive manner. Ultimately a light reliable embedded system will be built to help cardiologists in underdeveloped areas make the right decision quickly.

In summary, this study proposed light and intelligent system that uses deep learning for classifying heart valve disorders. The proposed system has been evaluated using augmented and non-augmented datasets of heart valve abnormalities. The major contributions of this study are as follows:

1. The use of a light CNN-LSTM model.
2. The use of augmented datasets for training and building a robust model.
3. The first to apply the CNN-LSTM architecture to discriminate heart valvular disorders.
4. Comparing the use of time domain and frequency domain inputs on the proposed model performance.
5. Comparing different deep learning models, the CNN model, the LSTM model, and the combined CNN-LSTM model.

The remainder of the paper is organized as follows. “Literature Review” section describes the related literature, and “Methods” section describes the dataset used in this study. “Results” section describes the proposed approach and the training procedure. “Discussion” section addresses the experimental findings, and finally, we conclude the article and outline the future research direction in the “Conclusions” section.

## Literature Review

Multiple researchers sought to discriminate various cardiovascular diseases using heart sound recordings. Several researchers employed machine learning and deep learning methods, particularly Convolutional Neural Networks (CNN) to accomplish this task. Despite the significant achievements in this field, many limitations like the small size of data, inefficient training methods, and the unavailability of accurate models continue to hinder advancements in this domain. The use of phonocardiogram (PCG) signals to detect cardiac abnormalities is the latest trend, some investigated publicly available datasets, while others used private in-house datasets. In this section, we survey the most recent and relevant heart sound classification literature.

In 2014, Sun et al.^11^ used a boundary curve diagnostic model that uses time and frequency features combined with a Support Vector Machine (SVM) classifier to diagnose the cardiac sounds, and distinguish between four cardiac problems with 94.7% accuracy. In 2018, Son and Kwon^12^ used Mel Frequency Cepstral Coefficients (MFCC) combined with Discrete Wavelet Transform (DWT) features as an input to the Support Vector Machine (SVM), Deep Neural Network (DNN), and K-Nearest Neighbor (KNN) classifiers, and they achieved an accuracy of 97.9%, 92.1%, and 97.4% respectively. In 2019, Alqudah^13^ classified nonsegmented heart sound signals using instantaneous frequency estimation statistical features. Principal Component Analysis (PCA) was used for dimensionality reduction, they achieved 91.6% for the K-Nearest Neighbor (KNN), and 94.8% for the Random Forest (RF) classifiers.

In 2020, Ghosh et al.^14^ used Deep Layer Kernel Sparse Representation Network (DLKSRN) classifier for the detection of different heart valve diseases using time–frequency representation of PCG recordings. Nonlinear features like L1-norm (LN), Sample Entropy (SEN), and Permutation Entropy (PEN) were extracted from the time–frequency matrix of the PCG recording, and they achieved a 99.24% accuracy. Alqudah et al.^15^ used AOCTNet architecture to discriminate between five different cardiovascular diseases using full bispectrum analysis of heart sound recordings and adaptive momentum optimization technique. They achieved a 98.7% accuracy for full images, and 96.1% for contour images. Ghosh et al.^16^ used the chirplet transform of the PCG cycle to propose a multiclass composite classifier that uses Local Energy (LEN) and Local Entropy (LENT) features extracted from the PCG signal in the time–frequency domain. They achieved 98.33% accuracy in discriminating between all four Valvular Heart Diseases (VHD) classes. Baghel et al.^17^ developed an automated system with low time complexity to discriminate various cardiac valve disorders from phonocardiograms using a Convolutional Neural Network (CNN). They used data augmentation, and a Gaussian filter for noise removal, the suggested model achieved an accuracy of 98.6% with augmented data, and 96.23% without data augmentation. Oh et al.^18^ classified heart sounds using a novel WaveNet model and achieved a 94% accuracy. They used 1000 PCG with 200 recordings per category, the model was validated using tenfold cross-validation and classified phonocardiogram (PCG) into five different classes.

In 2021, Alkhodari et al.^19^ utilized a CNN-BiLSTM network to discriminate five Valvular Heart Diseases (VHD) using phonocardiogram (PCG) recordings. The data were normalized and preprocessed using Maximum Overlap Discrete Wavelet Transform (MODWT), they achieved 99.32% accuracy, 0.998 Area Under the Curve (AUC), and 98.3 F1-score with tenfold cross-validation. Samiul Based Shuvo et al.^20^ developed a novel CardioXNet end-to-end architecture, based on a lightweight CRNN structure to discriminate the five Valvular Heart Diseases (VHD). The proposed architecture was fully automated and consisted of two-phase learning, the representation, and sequence residual learning phases, they achieved the highest reported accuracy of 99.6%, and a 99.4 F1 score.

## Methods

**Figure 1:**
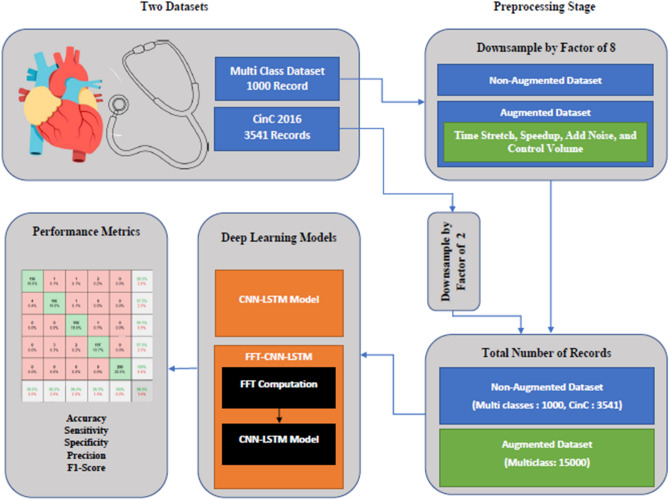
Block diagram of the proposed methodology.

The main objective of this research is to develop a new deep learning model based on the CNN-LSTM architecture to reliably distinguish heart sounds (binary and multi-class classifications). Figure 1 shows the block diagram of the proposed methodology, the following sub-sections describe in detail; the used datasets, the proposed methodology, and the performance metrics utilized to evaluate the suggested method.

### Datasets

**Figure 2:**
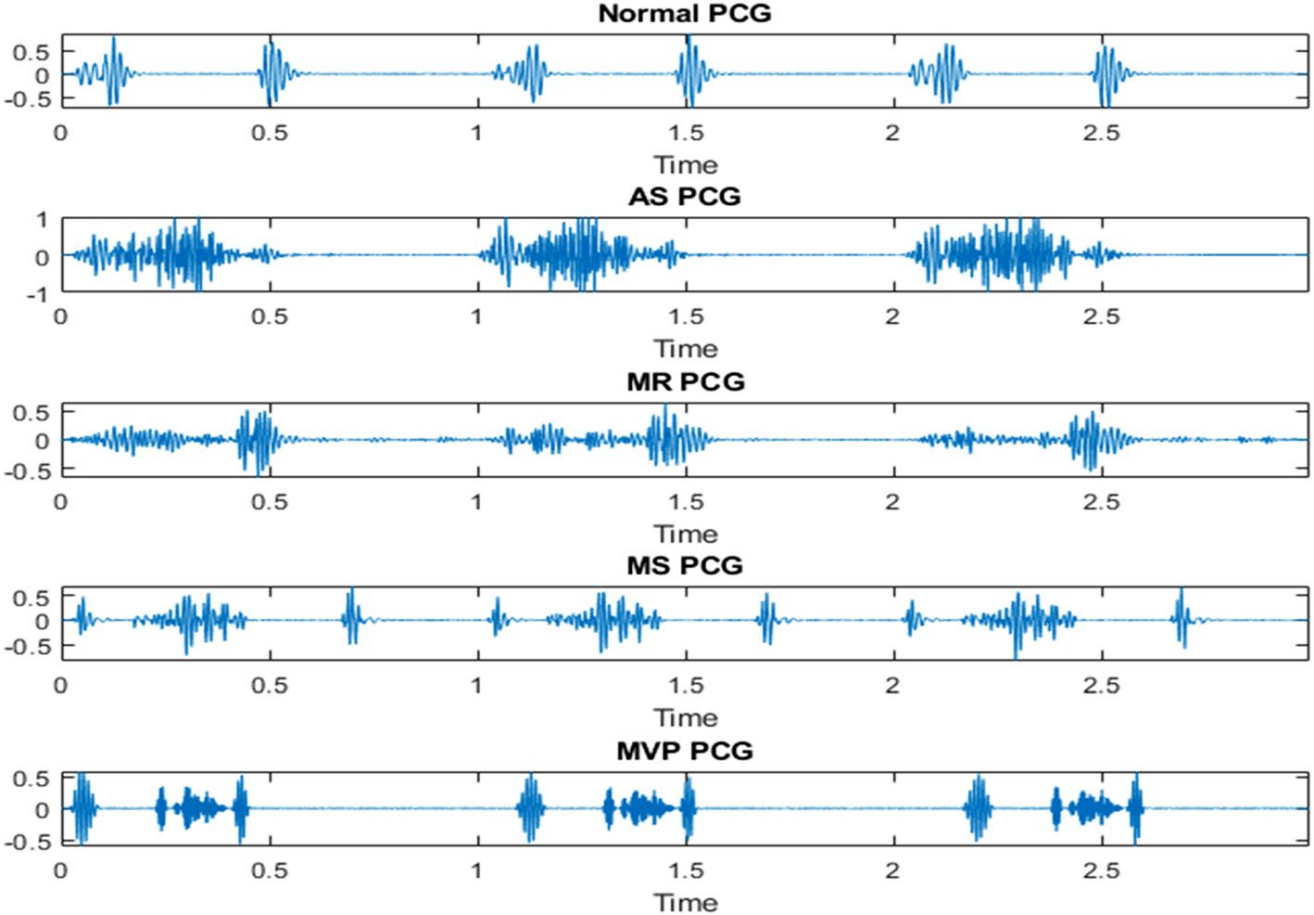
Sample PCG records from the first dataset.

The model was trained using the publicly available open heart sounds dataset^12^. The dataset contains 1000 audio clips gathered from various sources; the duration of each recording is nearly 3 s. As shown in Table 1, the data is divided into five categories with 200 clips in each category. The recordings are in *.wav audio format, were sampled at 8000 Hz, and converted to a mono channel format. The dataset contains five main classes which are the normal (N), aortic stenosis (AS), mitral stenosis (VS), mitral regurgitation (MR), and mitral valve prolapse (MVP). Table 1 summarizes the dataset being used, and Fig. 2 shows samples of different heart valve signals from the first dataset. All methods were performed following the relevant guidelines and regulations.

**Table 1:** The details of the primary dataset before augmentation.

Class	Number of records
Normal	200
AS	200
MS	200
MVP	200
MR	200

**Figure 3:**
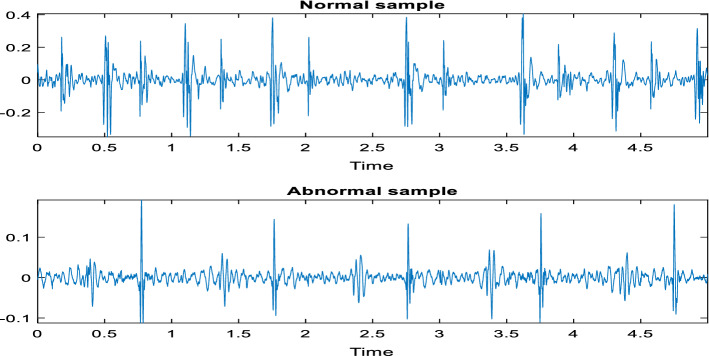
Sample PCG records from the second dataset.

PhysioNet/Computing in Cardiology Challenge 2016 was the second dataset utilized in this research to further examine the suggested model^13^. This dataset contains normal and abnormal classes only, all records have a sampling frequency of 2000 Hz and were converted to a mono channel format. Table 2 summarizes the dataset being used, and Fig. 3 shows samples of different heart valve signals from the second dataset. All methods were performed following the relevant guidelines and regulations.

**Table 2:** The details of the second dataset.

Class	Number of records
Normal	2725
Abnormal	816

### Fast Fourier transform (FFT)

**Figure 4:**
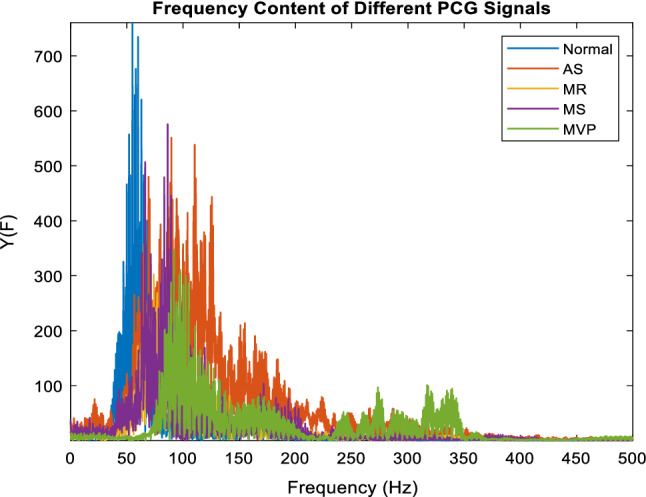
The frequency content of five PCG classes.

A fast Fourier transform (FFT) is a method that computes a signal’s discrete Fourier transform (DFT) or its inverse (IDFT). Fourier analysis transforms a signal from its native time domain to a frequency domain representation and vice versa. Decomposing a series of values into components with various frequencies yields the DFT^21^. Computing this operation straight from the definition is frequently too slow, by dividing the DFT matrix into a product of sparse elements, an FFT can perform such modifications quickly^22^. The performance difference can be substantial, especially for large data sets with N in the hundreds of millions^23^. Fast Fourier transformations are commonly utilized in engineering, music, science, and mathematics. Although the fundamental principles were popularized in 1965, several algorithms had been developed as early as 1805. Gilbert Strang referred to the FFT as "the most important numerical algorithm of our lifetime" in 1994, and it was named one of the IEEE journal Computing in Science & Engineering's Top 10 Algorithms of the 20th Century^24^. In this paper, the Fourier transform of PCG signals was clipped to contain only 350 Hz from the 4000 Hz spectrum; this is because the major components are in this frequency range^16^. Figure 4 shows the whole spectrum of five different PCG signals.

### Down sampling

Earlier studies^16,25^ show that the maximum frequency component content in the PCG signal is around 300 Hz, accordingly, the selected down sampling frequency of 1 kHz is sufficient to represent the PCG intrinsic data. To make the classification process faster and more accurate, each PCG record in the first dataset is downsampled by a factor of 8, and each PCG record in the second dataset is downsampled by a factor of 2. These factors were obtained from previous studies like^16,26^, and^27^, and they are sufficient to describe the frequency content of the whole signal. Figure 4 shows that the highest frequency content is 500 Hz in all heart conditions.

### Data augmentation

Data augmentation is a popular technique used to artificially enlarge the size of a given dataset^27^. In general, augmentation attempts to generate various versions of the audio clips by applying diverse enlargement techniques^28^. Moreover, training deep learning systems on large datasets makes them more skillful at dealing with different version of inputs that resemble real-life inputs, as a result, the augmentation techniques creates a variation in the audio files that results in a better overall performance^29,30^. Similar to images, there are several techniques to augment audio signals, and these techniques are usually applied to the raw audio signals^30,31^. Table 3 summarizes the primary dataset after augmentation. In this research, the following audio augmentation techniques were applied:

- Time stretch: randomly slow down or speed up the sound.
- Time shift: shift audio to the left or the right by a random amount.
- Add noise: add some random values to the sound.
- Control volume: randomly increasing or decreasing the volume of the audio.

**Table 3:** The details of the primary dataset after augmentation.

Class	Number of records
Normal	3000
AS	3000
MS	3000
MVP	3000
MR	3000

### Deep learning CNN-LSTM model

**Figure 5:**
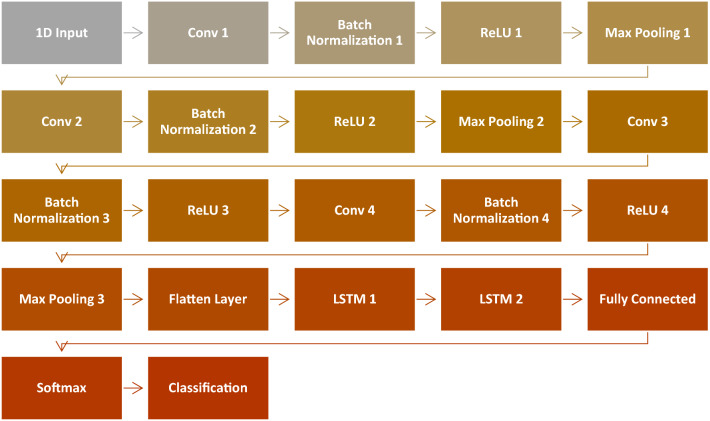
The proposed CNN-LSTM Model.

Deep learning is the most recent and cutting-edge machine learning method employed in response to the expanding number of large datasets^32–36^. Deep learning is based on and inspired by the deep structure of the human brain^37,38^. The architecture of the human brain has a huge number of hidden layers, allowing us to extract and abstract deep information at different levels and from different perspectives. Deep learning is concerned with the development of a specialized architecture comprised of multiple and sequential layers in which successive phases of input processing are conducted^38^. A plethora of deep learning structures have been proposed in recent years^34,39^, Convolutional Neural Network (CNN)^39,40,41^ and Long Short-Term Memory (LSTM)^42–45^ are the most known, widely used, and efficient deep learning algorithms. The proposed hybrid CNN-LSTM model is described in Fig. 5. Deep feature extraction and selection from the PCG signals are handled by CNN blocks, particularly the 1D convolutional layers, the batch normalization layers, the ReLU layers, and the max-pooling layers. Whilst the LSTM module extracts contextual time data after being fed these qualities as time-dependent features^46^. Studies suggest that deep feature extraction and classification using a hybrid 1D CNN-LSTM outperforms single CNN or LSTM-based approaches^47,48^. Furthermore, utilizing the LSTM component produce a richer and more concentrated model compared to the pure CNN models, resulting in higher performance with fewer parameters. Table 4 shows the detailed description of the layers in the proposed CNN-LSTM architecture.

**Table 4:** The proposed CNN-LSTM architecture.

Layer number	Layer name	Layer description
1	1D input	Size 8000 for time input and 1000 for FFT input
2	Conv 1	Number of filters: 48; kernel size: 3 × 1
3	Batch normalization 1	Number of channels: 48
4	ReLU 1	–
5	Max pooling 1	Kernel size: 2 × 1; stride: 2
6	Conv 2	Number of filters: 32; kernel size: 3 × 1
7	Batch normalization 2	Number of channels: 32
8	ReLU 2	–
9	Max pooling 2	Kernel size: 2 × 1; stride: 2
10	Conv 3	Number of filters: 16; kernel size: 3 × 1
11	Batch normalization 3	Number of channels: 16
12	ReLU 3	–
13	Conv 4	Number of filters: 64; kernel size: 3 × 1
14	Batch normalization 4	Number of channels: 64
15	ReLU 4	–
16	Max pooling 3	Kernel size: 2 × 1; stride: 2
17	Flatten layer	–
18	LSTM 1	Number of hidden units: 64
19	LSTM 2	Number of hidden units: 32
20	Fully connected	Output: 5
21	Softmax	–
22	Classification	–

### Ablation study

The goal of this section is to explore what makes our model light and different from other models. In this section, we study the robustness of the network performance against the structural changes caused by ablations, as some layers are removed or added^49^. The ablation study removed the LSTM and CNN components from the model and analyzed the effect of removing them on the model performance. The ablations to the suggested CNN-LSTM model had both negative and positive effects on the classification performance^49^. The greater the number of ablated layers, the more powerful the impact on performance. The study found that various layers have various impacts on classification performance^50^. Finally, the ablation study concluded that the performance of the proposed CNN-LSTM model is higher than any single model and this combination of components resulted in the highest performance ever.

### Model evaluation

**Figure 6:**
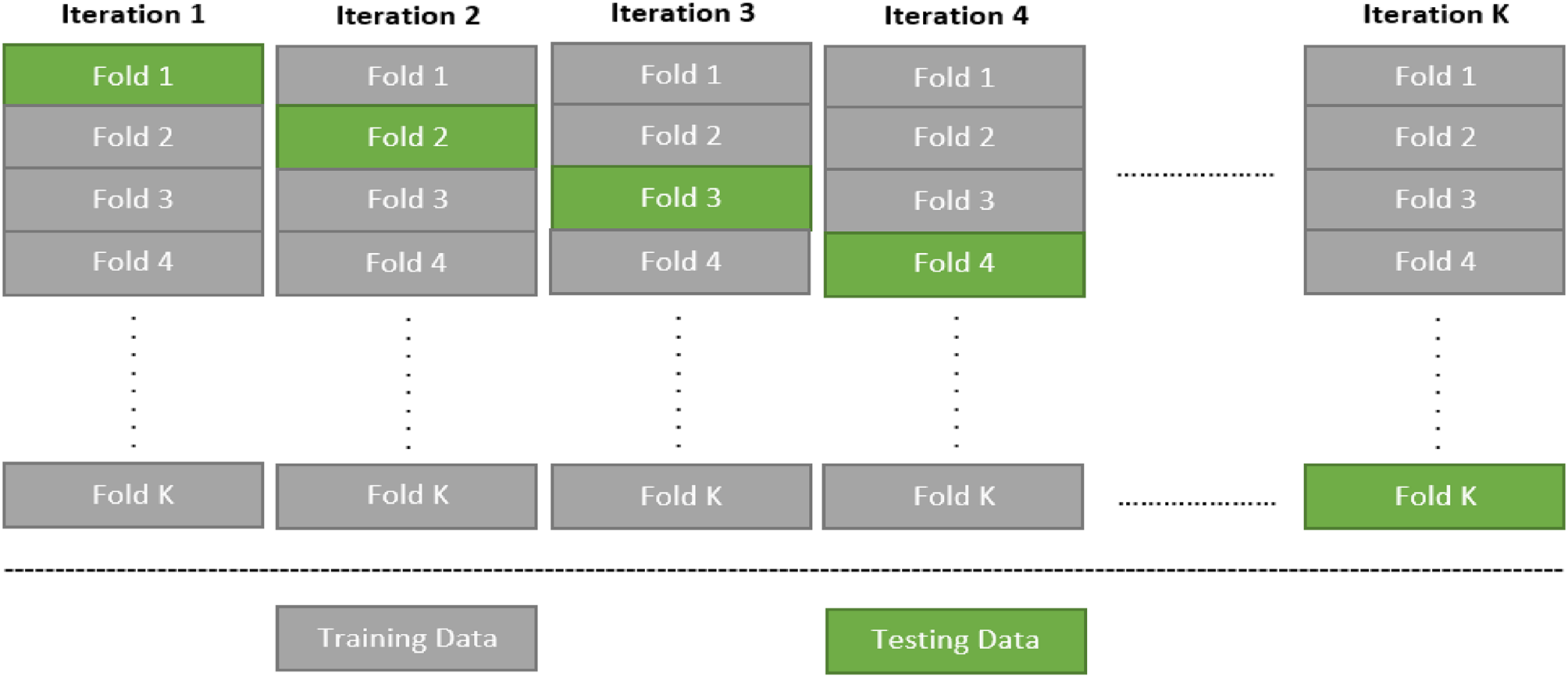
Block diagram of K-fold cross-validation.

In general, evaluating any machine learning or deep learning model is a challenging task due to varying dataset sizes. Typically, machine learning engineers divide the data into training and testing sets with different ratios, they use the training set to train the model and the testing set to assess the model. Although this validation technique is appropriate when the dataset is large, it is not reliable because the accuracy obtained for one test set can be very different from the accuracy obtained using another^35,43^. The K-fold Cross-Validation provides an ideal answer to this problem, the solution is to divide the data into folds ensuring that each fold serves as a testing set at some point. In this study, tenfold cross-validation was used to evaluate the model, it guarantees that the model generalized properly, and it also helps prevent overfitting. Finally, different performance metrics were calculated to evaluate the performance of the proposed model^34,43^. Figure 6 illustrates the k-fold cross-validation methodology.

### Performance metrics

To evaluate the performance of the proposed methodology in classifying heart valve anomalies, the confusion matrix for the binary classification and multi-class classification (with and without augmentation) tasks were calculated. The outcomes of the CNN-LSTM model were compared to the corresponding label of the original PCG signal^16^. Using the resulting confusion matrix, four statistical indices were calculated and utilized to measure the performance of the suggested system, namely True Positive (TP), False Positive (FP), False Negative (FN), and True Negative (TN). Based on these statistical values, accuracy, sensitivity, specificity, and the F1-Score metrics were calculated.

1 $$\mathrm{Accuracy}= \frac{\mathrm{TP}+\mathrm{TN}}{\mathrm{TP}+\mathrm{FP}+\mathrm{TN}+\mathrm{FN}}$$

2 $$\mathrm{Sensitivity}= \frac{\mathrm{TP}}{\mathrm{TP}+\mathrm{FN}}$$

3 $$\mathrm{Specificity}= \frac{\mathrm{TN}}{\mathrm{TN}+\mathrm{FP}}$$

4 $$\mathrm{Precision}= \frac{\mathrm{TP}}{\mathrm{FP}+\mathrm{TP}}$$

5 $$\mathrm{F}1{\text{-}}\mathrm{Score}=2*\frac{\mathrm{Precision}*\mathrm{Sensitivity}}{\mathrm{Precision}+\mathrm{Sensitivity}}$$

To further evaluate the proposed CNN-LSTM model performance, the Receiver Operating Characteristics (ROC) curve was generated, and Area Under Curve (AUC) was also calculated to give a quantitative estimation.

## Results

In this section, the effectiveness of the proposed CNN-LSTM Model is evaluated using several performance metrics. As explained, the suggested CNN-LSTM model is the result of employing extensive ablation studies using single CNN and LSTM models. All the experiments were conducted on a desktop computer that runs Microsoft Windows, utilizes an Intel Core i7-6700/3.4 GHz processor, 16 GB of RAM, and a 500 GB hard disk drive (HDD). The tenfold methodology was used to test the proposed model, and one of the 9 folds used for training was used as validation during the cross-validation process. the Adam optimizer, and the cross-entropy loss function^37,38^ were employed for each loss function. The following sections will illustrate the results of the ablation study together with the proposed model.

### Ablation study

**Figure 7:**
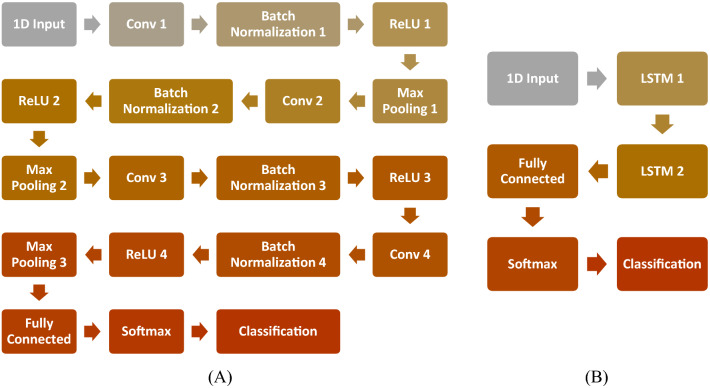
(**A**) CNN and (**B**) LSTM model architectures.

The ablation study conducts various element changes in the base architecture, the cross-validation accuracy is calculated for each experimental configuration, and the results are reported. In the first case study, we use the CNN model without any LSTM layers, while in the second case study, we use the LSTM model without any CNN layers. Figure 7 shows the two suggested model architectures.

**Figure 8:**
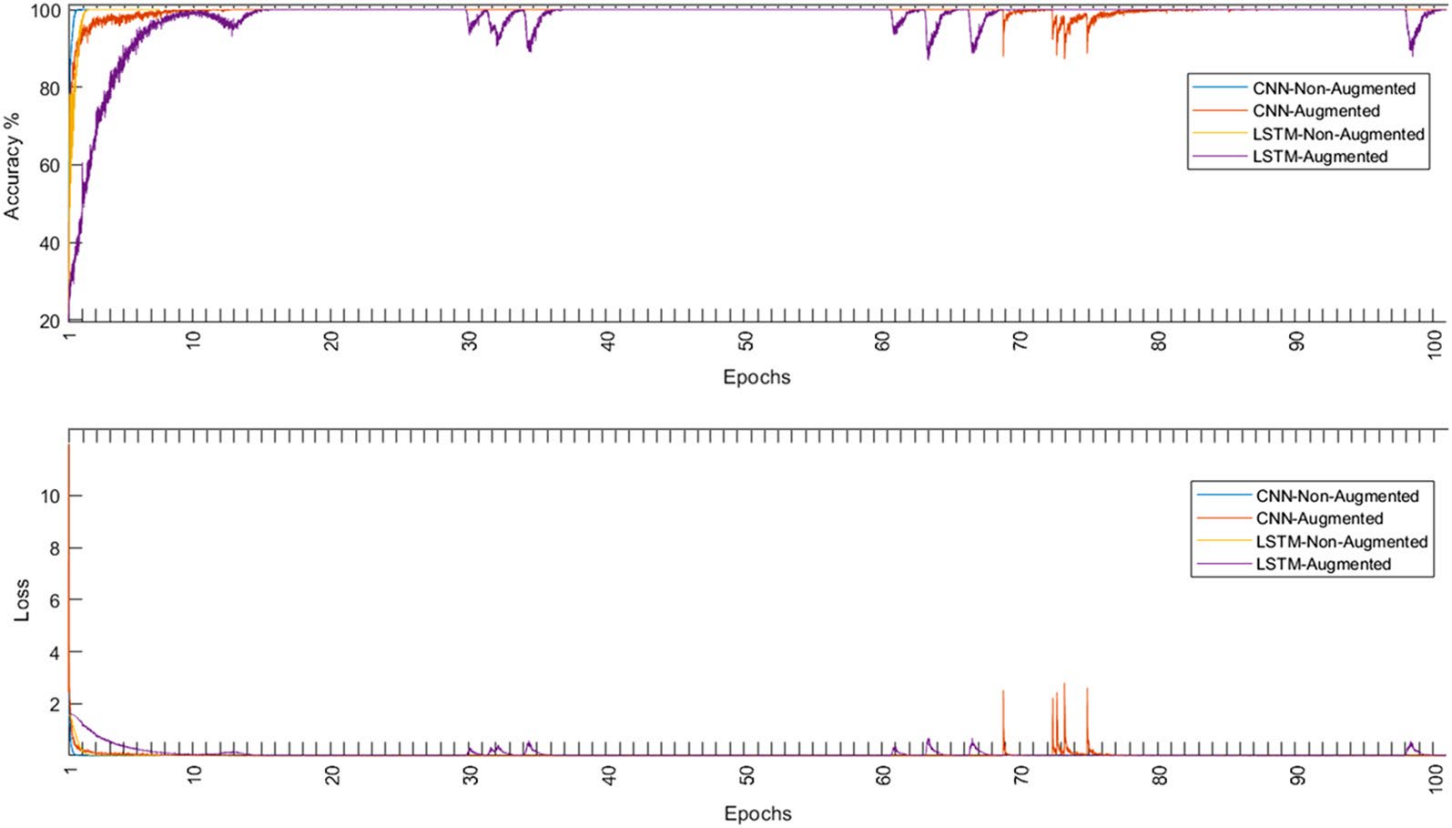
The average training accuracy and loss curves of different folds for all models in the ablation study.

Both models were evaluated using the augmented and non-augmented datasets, the tenfold cross-validation methodology was used to test the proposed models, the Adam optimizer, and the cross-entropy loss function^37,38^ were employed for each loss function. Using an initial learning rate of 0.001, the suggested models were trained for 100 max epochs per fold. The combination of these hyperparameters resulted in the best performance for each model. Table 5 shows the performance metrics of the models in the ablation study while Fig. 8 shows the average training and loss curves among all folds of different models.

**Table 5:** Ablation study using different model architectures.

Dataset	Model			
	CNN only		LSTM only	
	Non-augmented	Augmented	Non-augmented	Augmented
Accuracy	94.60	97.96	50.70	71.07
Sensitivity	94.50	97.96	50.70	71.07
Specificity	98.63	99.94	87.68	92.77
Precision	94.54	97.96	53.69	71.75
F1-score	94.50	97.96	51.64	71.30

After completing the ablation studies on the two basic models (CNN and the LSTM), the proposed CNN-LSTM model is constructed by combining both of these models, and a significant improvement in classification performance was observed. The configuration of the CNN-LSTM model will be discussed in the next section.

### Proposed CNN-LSTM model

**Figure 9:**
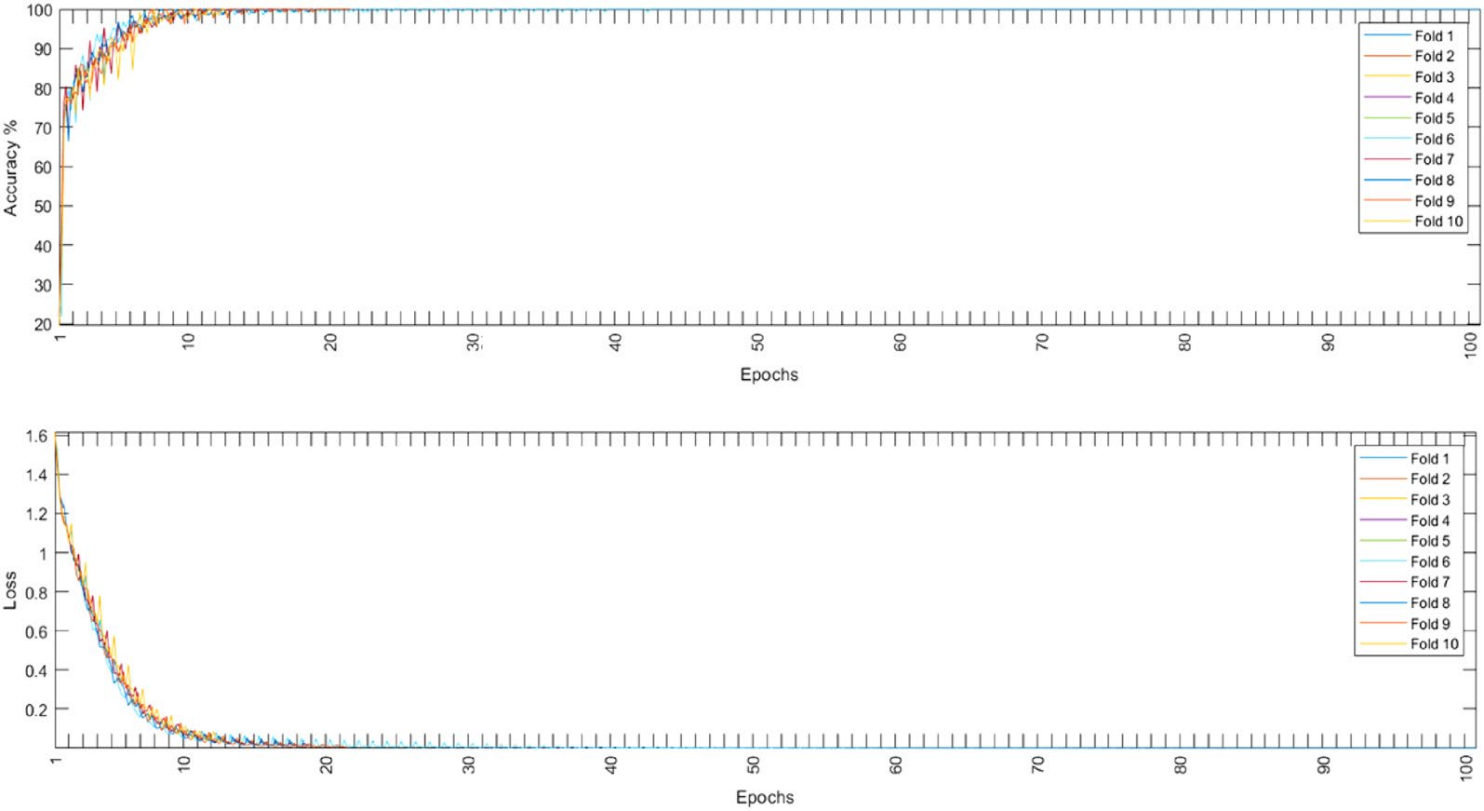
Non-augmented training accuracy and loss.

**Figure 10:**
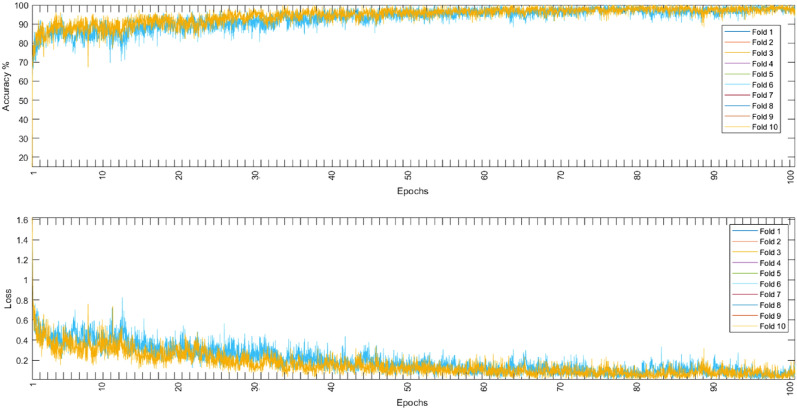
Augmented training accuracy and loss.

**Figure 11:**
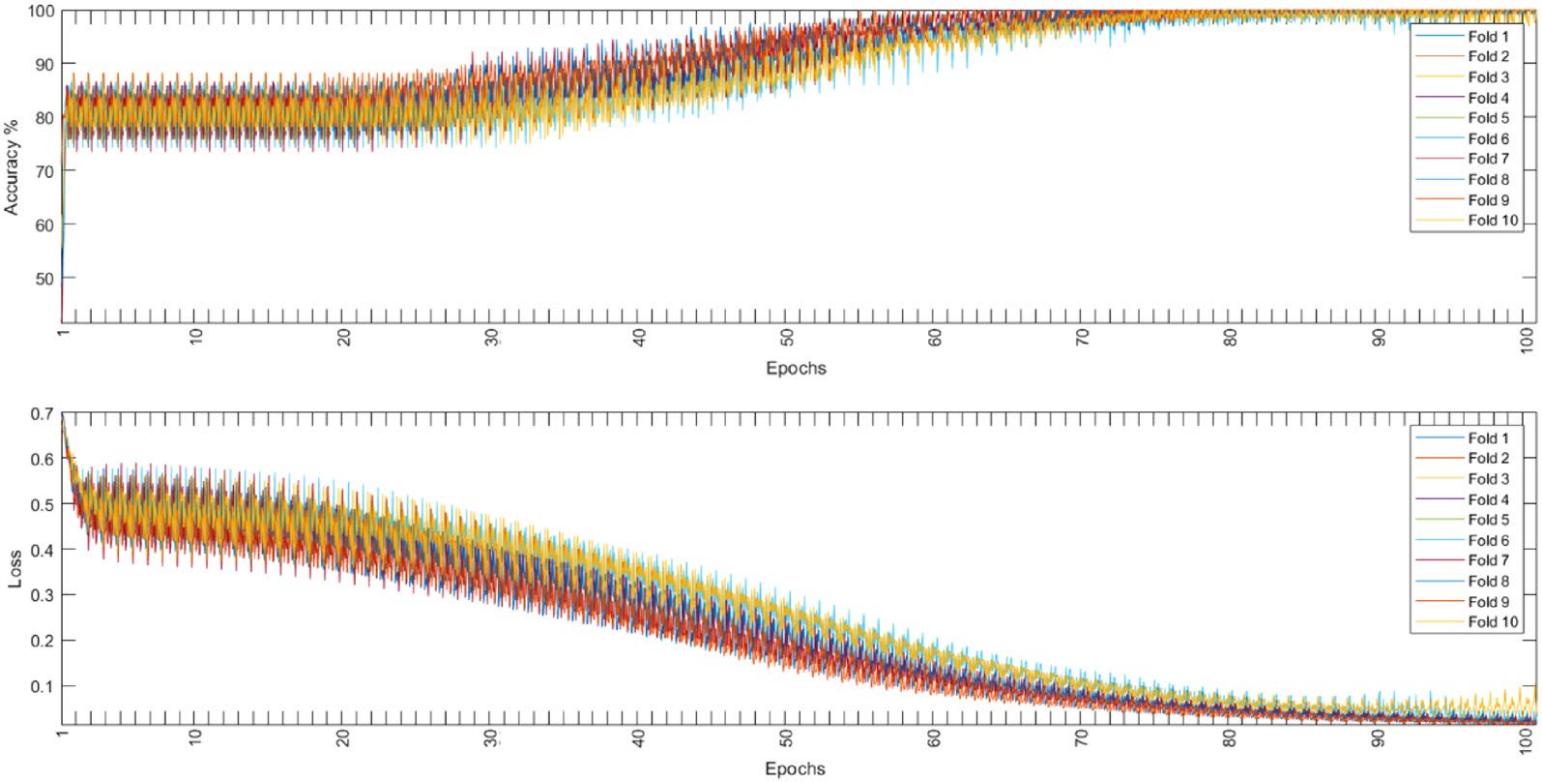
Binary dataset training accuracy and loss.

The initial learning rate was 0.001 for time domain inputs training and 0.0001 for frequency domain inputs, using these values, the suggested architecture was trained for 100 max epochs per fold. Figures 9, 10, and 11 show the training accuracy and loss for all folds among non-augmented, augmented, and binary classification respectively.

**Figure 12:**
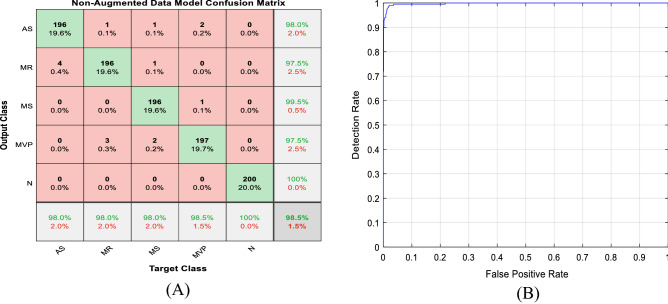
(**A**) Non-augmented data model confusion matrix. (**B**) Receiver operating characteristic (ROC) curve.

**Figure 13:**
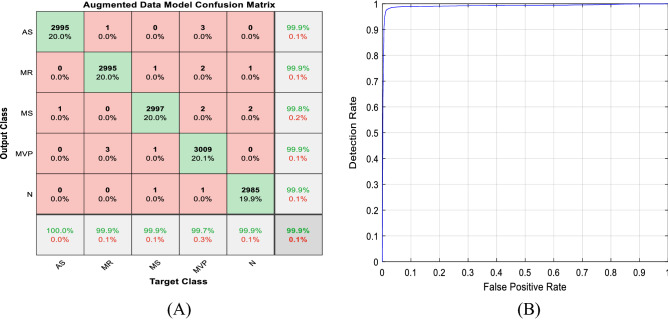
(**A**) Augmented data model confusion matrix. (**B**) Receiver operating characteristic (ROC) curve.

**Figure 14:**
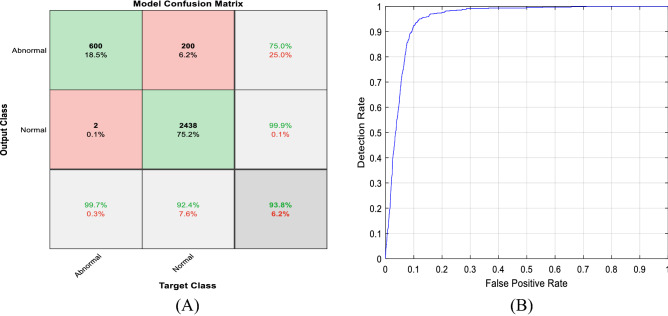
(**A**) Binary classification data model confusion matrix. (**B**) Receiver operating characteristic (ROC) curve.

The first part of Figs. 12, 13, 14 shows the five class confusion matrix using the non-augmented and augmented data respectively. The rows represent the actual class, whereas the columns represent the predicted class. In the case of non-augmented data, the accuracy is 98.5%, with a small number of incorrect classifications measured by the number of False Positives (FP) and False Negatives (FN) of 1.5%. For the augmented data, the accuracy is 99.9%, and the number of False Positives (FP) and False Negatives (FN) is 0.1%. It is clear from both figures that increasing the size of the dataset using different augmentation techniques increased accuracy by 1.4% to near 100% and lowered incorrect predictions by 1.4% to 0.1%.

The second part of Figs. 12, 13, 14 displays the Receiver Operating Characteristic (ROC) curves for the augmented and non-augmented data. The ROC is a visual way to represent the tradeoff between specificity and sensitivity, it plots the True Positive Rate (TPR) against the False Positive Rate (FPR) at various threshold settings. It is obvious from both figures that the curve is close to the upper left corner indicating the excellent model diagnostic ability, it is also apparent from the figures that the Area Under the Curve (AUC) for the augmented data is slightly better than that of the non-augmented data. Figure 14 shows the confusion matrix, and the Receiver Operating Characteristic (ROC) curve for the binary (normal/abnormal) classification problem. Accuracy is 93.8%, and Area Under the curve (AUC) is 0.9505 indicating high performance. The drop in accuracy between the binary and multiclass class problem can be attributed to the larger size of the PhysioNet/CinC 2016 challenge dataset (5878 vs. 1000 audio files).

In this paper, PCG signals were classified into 5 different classes using the augmented or the non-augmented version of the open heart sounds dataset or into two categories using the PhysioNet/CinC 2016 challenge dataset. The proposed CNN-LSTM architecture exhibited very high performance for all important metrics, it achieved near-perfect accuracy on the given datasets using 10-folds cross-validation. Tables 6, 7, and 8 show the accuracy, sensitivity, specificity, precision, and F1 scores for all experiments conducted. Figures 9, 10, and 11 show that the suggested model converged rapidly reaching 100% training accuracy quickly.

**Table 6:** Results of tenfold cross-validation using non-augmented dataset.

Fold	Accuracy	Sensitivity	Specificity	Precision	F1-Score
1	98.35	98.31	99.52	98.75	98.30
2	98.51	98.51	99.76	98.56	98.52
3	98.29	98.60	99.59	98.54	98.63
4	98.31	98.69	99.78	98.78	98.95
5	98.50	98.77	99.49	98.36	98.38
6	98.77	98.52	99.38	98.63	98.53
7	98.37	98.24	99.38	98.65	98.49
8	98.56	98.37	99.71	98.46	98.16
9	98.46	98.31	99.59	98.54	98.42
10	98.70	98.91	99.59	98.30	98.19
Mean	98.482 (± 0.16)	98.523 (± 0.22)	99.579 (± 0.14)	98.557 (± 0.15)	98.457 (± 0.23)

**Table 7:** Results of tenfold cross-validation using augmented dataset.

Fold	Accuracy	Sensitivity	Specificity	Precision	F1-Score
1	99.88	99.85	99.93	99.89	99.83
2	99.83	99.94	100.03	99.82	99.91
3	99.91	99.83	100.02	99.83	99.88
4	99.88	99.85	99.96	99.84	99.87
5	99.88	99.84	99.91	99.85	99.88
6	99.89	99.82	99.95	99.86	99.81
7	99.88	99.86	99.96	99.87	99.87
8	99.83	99.86	99.98	99.75	99.93
9	99.86	99.93	99.96	99.85	99.87
10	99.86	99.86	99.99	99.92	99.87
Mean	99.87 (± 0.03)	99.864 (± 0.04)	99.969 (± 0.04)	99.848 (± 0.05)	99.872 (± 0.03)

**Table 8:** Results of tenfold cross-validation using binary dataset.

Fold	Accuracy	Sensitivity	Specificity	Precision	F1-score
1	93.69	99.76	92.45	97.41	85.59
2	93.76	99.49	92.53	98.02	85.4
3	93.78	99.6	92.52	97.70	85.69
4	93.8	99.63	92.35	97.59	85.68
5	93.72	99.70	92.45	97.70	85.59
6	93.74	99.49	92.33	97.57	85.58
7	93.96	99.71	92.29	97.58	85.34
8	93.53	99.54	92.51	97.81	85.65
9	93.98	99.67	92.42	97.80	85.37
10	93.79	99.73	92.41	97.80	85.36
Mean	93.775 (± 0.13)	99.632 (± 0.1)	92.426 (± 0.08)	97.698 (± 0.17)	85.525 (± 0.14)

Table 9 shows the various performance metrics of the different examined datasets. For the non-augmented data, the accuracy was 98.5%, sensitivity was 98.5%, specificity was 99.625%, precision was 98.505%, F1-score was 98.5%, and Area Under the Curve (AUC) was 0.997. For the augmented data, the accuracy was 99.87%, sensitivity was 99.87%, specificity was 99.96%, precision was 99.87%, F1-score was 99.87%, and Area Under the Curve (AUC) was 0.998. For the binary dataset, the accuracy was 93.77%, sensitivity was 99.63%, specificity was 92.42%, precision was 97.6%, F1-score was 85.52%, and Area Under the Curve (AUC) was 0.95. It is clear from the table that the augmented data outperforms the non-augmented data for all performance metrics. It is also noticeable that using the augmented data, the proposed hybrid model achieved a near 100% accuracy. Table 10 displays the performance metrics obtained for each condition using the multiclass dataset. It is clear from the table that the suggested model exhibited very high precision and recall scores for all the tested classes.

**Table 9:** Comparison of the average performance metrics of different datasets.

Metric	Non-augmented	Augmented	Binary
Accuracy	98.482	99.87	93.775
Sensitivity	98.523	99.864	99.632
Specificity	99.579	99.969	92.426
Precision	98.557	99.848	75.00
F1-score	98.457	99.872	85.525
AUC	0.9978	0.9985	0.9505

**Table 10:** Comparison of the performance metrics of augmented and non-augmented datasets.

Data	Non-augmented			Augmented		
	Pre	Sen	F1	Pre	Sen	F1
AS	98 (± 0.2)	98 (± 0.2)	98 (± 0.2)	99.86 (± 0.0.01)	99.979 (± 0.0.01)	99.92 (± 0.0.01)
MR	97.51 (± 0.2)	98 (± 0.0.1)	97.75 (± 0.1)	99.87 (± 0.0.01)	99.87 (± 0.0.01)	99.87 (± 0.0.01)
MS	99.49 (± 0.05)	98 (± 0.2)	98.74 (± 0.2)	99.83 (± 0.0.01)	99.90 (± 0.0.01)	99.87 (± 0.0.01)
MVP	97.52 (± 0.2)	98.50 (± 0.1)	98.01 (± 0.1)	99.87 (± 0.0.01)	99.73 (± 0.0.01)	99.80 (± 0.0.01)
N	100 (± 0.0)	100 (± 0.0)	100 (± 0.0)	99.93 (± 0.0.01)	99.89 (± 0.0.01)	99.92 (± 0.0.01)

*Pre* precision, *Sen* sensitivity, *F1* F1-score.

### Result of testing the proposed CNN-LSTM model using FFT inputs

**Figure 15:**
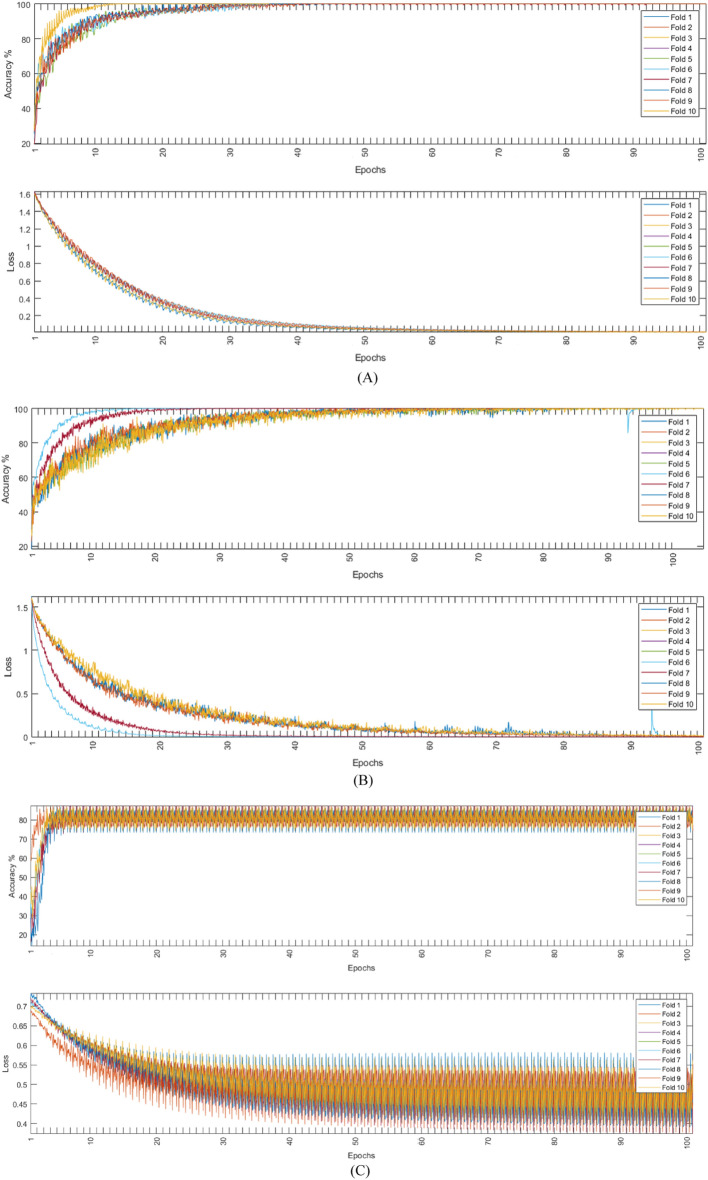
Augmented training accuracy and loss; (**A**) for non-augmented dataset, (**B**) augmented dataset, (**C**) binary dataset.

**Figure 16:**
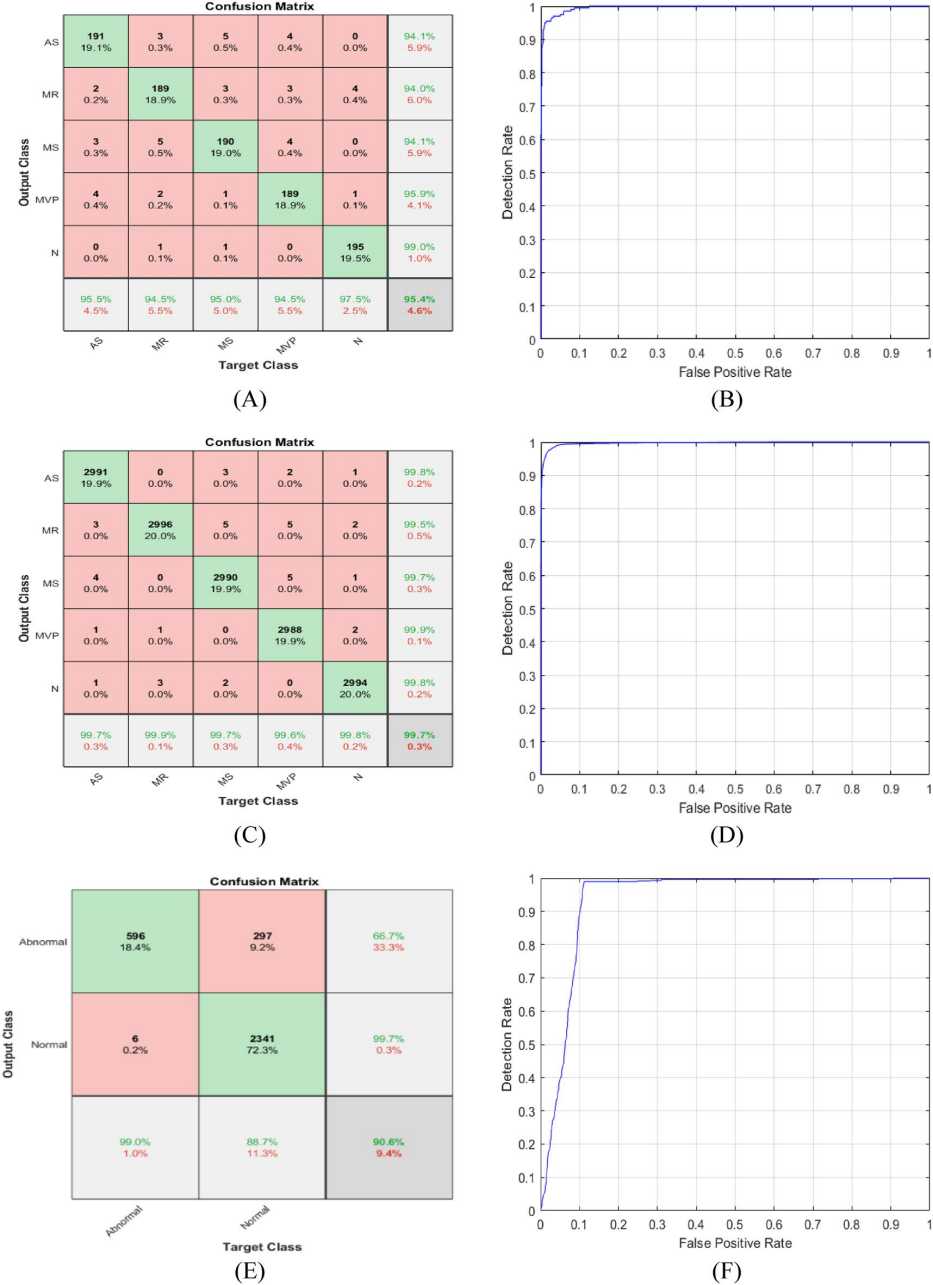
(**A**) Non-Augmented confusion matrix, (**B**) non-augmented receiver operating characteristic (ROC) curve. (**C**) augmented confusion matrix, (**D**) augmented receiver operating characteristic (ROC) curve. (**E**) binary confusion matrix, (**F**) binary receiver operating characteristic (ROC) curve.

To further investigate the performance of the proposed CNN-LSTM model, the suggested model was modified to accept inputs from the frequency domain (FFT), as a result, the size of the input layer changed to [1000 × 1]. Table 11 shows the various performance metrics of the different examined datasets using the FFT-CNN-LSTM model. For the non-augmented data, the accuracy was 95.40%, sensitivity was 95.40%, specificity was 98.85%, precision was 95.42%, F1-score was 95.41%, and Area Under the Curve (AUC) was 0.9963. For the augmented data, the accuracy was 99.73%, sensitivity was 99.73%, specificity was 99.93%, precision was 99.73%, F1-score was 99.73%, and Area Under the Curve (AUC) was 0.9971. It is clear from the table that the use of augmented data was better than the use of non-augmented data for all performance metrics. It is also noticeable that using the augmented data, the proposed hybrid model achieved a near 100% accuracy. Figure 15 shows the training accuracy and loss for all folds among non-augmented, augmented, and binary datasets respectively. While Fig. 16 shows the non-augmented, and binary datasets confusion matrix and ROC curves using the FFT-CNN-LSTM.

**Table 11:** Comparison of the average performance metrics of different datasets.

Metrics	Non-augmented	Augmented	Binary
Accuracy	95.40	99.73	90.65
Sensitivity	95.40	99.73	99.00
Specificity	98.85	99.93	88.74
Precision	95.42	99.73	66.74
F1-score	95.41	99.73	79.73
AUC	0.9963	0.9971	0.9367

**Figure 17:**
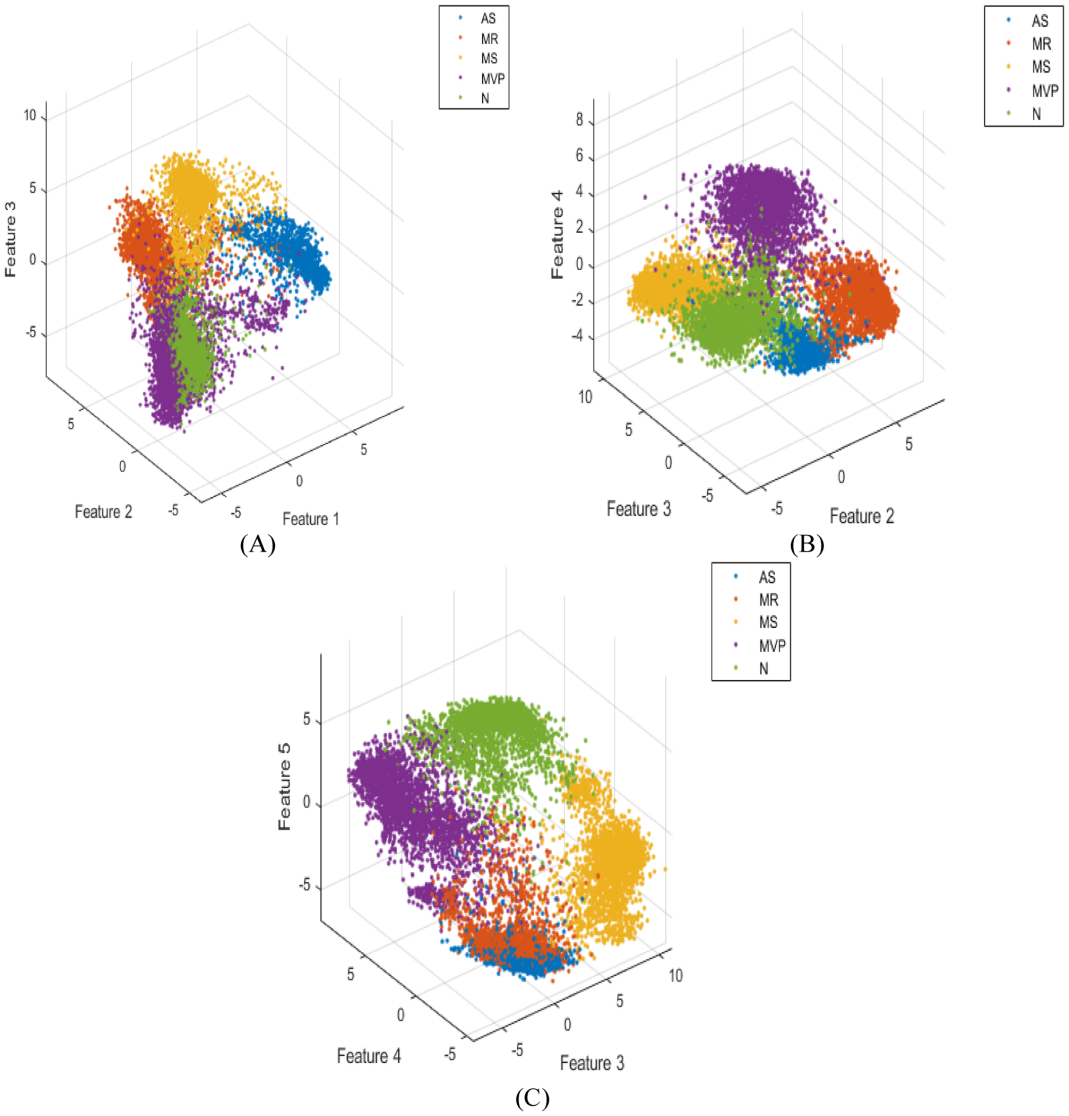
The extracted features from the last fully connected layer of the proposed model; (**A**) using features 1, 2, and 3. (**B**) using features 2, 3, and 4. (**C**) using features 3, 4, and 5.

To evaluate if the deep features extracted using the proposed CNN-LSTM were significant, discriminant, and representative in the classification of different heart sounds, a scatter plot of the extracted deep features among five classes was drawn from the last fully connected layer of the proposed model. It can be noticed from Fig. 17 that the range of different extracted features among different classes was far off from each other, which means that the extracted features can be used successfully in the classification of heart valve diseases. Also, it can be concluded from Fig. 17 that each extracted feature was representative of its class and that managed to discriminate it from other classes.

## Discussion

Table 12 summarizes the performance metrics for the suggested model and compares it to the recent and relevant state-of-the-art literature. All the models in the table use the same open heart sounds dataset^12^ that this research used but for Sun et al.^11^. It is clear from the table that the proposed architecture outperforms all models for all important performance metrics. The accuracy of the new model is 99.87% which is 0.27% higher than the accuracy of the second-best model built by Shuvo et al.^20^ in 2021. It is also clear from Table 10 that augmenting the data has a positive impact since it improved accuracy by 1.37%. This result corroborates the findings obtained by Baghel et al.^17^ reported in 2020. In their case, augmenting the data improved model accuracy by 2.43%.

**Table 12:** Comparison between related work and the proposed method using the open heart sounds dataset.

Reference	Method	Acc		Sen	Spe	AUC
Sun et al.^11^	Time–frequency domain with SVM	94.8		–	–	–
Son and Kwon^12^	SVM	97.9		98.2	99.4	–
	DNN	92.1		94.5	98.2	–
	KNN	97.4		97.6	98.8	–
Alqudah et al.^13^	RF	94.8		94.78	–	–
	KNN	91.6		91.5		
Ghosh et al.^14^	DLKSRN classifier	99.24		–	–	–
Alqudah et al.^15^	CNN with bispectrum images	Full images	98.7	98.7	–	–
		Contour images	97.1	97.1	–	–
Ghosh et al.^16^	Multiclass composite classifier	98.33		98.33	–	–
Baghel et al.^17^	CNN	Augmentation	98.6	–	–	–
		Without augmentation	96.23			
Oh et al.^18^	WaveNet architecture	94		92.5	98.1	–
Alkhodari et al.^19^	CNN-BiLSTM	99.32		98.3	99.58	0.9980
Samiul based Shuvo et al.^20^	CRNN	99.60		99.52	–	–
Suggested model	CNN + LSTM in time domain	With augmentation	99.87	99.864	99.969	0.999
		Without augmentation	98.482	98.523	99.579	0.998
		Binary classes	93.775	99.632	92.426	0.951
	CNN + LSTM in frequency domain	With augmentation	99.73	99.73	99.93	0.997
		Without augmentation	95.40	95.40	98.85	0.996
		Binary classes	90.65	99.00	88.74	93.67

To further test the architecture generalization proficiency, the model was trained and tested on the widely used PhysioNet/CinC 2016 challenge dataset. Again, the raw data was used to train the new architecture; no data filtering, denoising, or augmentation techniques were applied. The obtained results displayed in Table 13 show that the system succeeded in discriminating between normal and abnormal cases with 93.76% accuracy, 99.66% sensitivity, 92.42% specificity, and an average Area Under the Curve (AUC) of 0.9505. The findings show that the new system outperformed the previous state-of-the-art models for all performance metrics. The obtained accuracy is 6.45% higher than the 87.31% accuracy reported by Alkhodari et al. in 2021^19^. The reason for the weak performance of the previous models can be attributed to the unbalanced nature of the PhysioNet/CinC 2016 challenge dataset that uncovered model weaknesses in generalizing properly.

**Table 13:** Comparison between related work and the proposed method using PhysioNet/CinC 2016 challenge dataset.

Authors	Method	Acc	Sen	Spe	AUC	F1-score
Alkhodari et al.^19^	CNN-BiLSTM	87.31	92.78	79.48	0.900	–
Samiul based Shuvo et al.^20^	CRNN	86.57	90.33	–	–	91.78
Suggested model	Time domain CNN + LSTM	93.775	99.632	92.426	0.951	85.525
	Frequency domain CNN + LSTM	90.65	99.00	88.74	0.9367	79.73

The proposed model performed effectively on both datasets, and the accuracy obtained in this research is almost perfect (nearly 100%) which makes the suggested architecture dependable and trustworthy. To the best of our knowledge, this is the highest accuracy ever reported in the literature. This model will have a positive impact on public health, building an embedded mobile system using this model can help physicians in rural areas detect cardiovascular problems early, quickly, accurately, and cost-effectively. This will help alleviate fatal complications, remove interpretation subjectivity and variability, and will also improve the health situation in remote regions that lack expert doctors by helping novice doctors in these areas make the right decision.

Using the FFT input, the FFT-CNN-LSTM model performance was efficient using both datasets, and the accuracy obtained using the FFT-CNN-LSTM model was 99.73% which makes using the frequency domain input dependable and trustworthy. The accuracy obtained using the time domain input was 99.83% slightly higher than the accuracy obtained using the frequency domain input which is 99.73%. To further test the system learning capability using the FFT input, the model was trained and tested on the widely used PhysioNet/CinC 2016 challenge dataset. Here, the raw data was used to train the new architecture; no preprocessing was applied. The system succeeded in discriminating between normal and abnormal cases with 90.65% accuracy, 99.00% sensitivity, 88.74% specificity, and an average Area Under the Curve (AUC) of 0.9367. This model also outperformed the state-of-the-art models for all performance metrics. The obtained accuracy is 3.34% higher than the 87.31% accuracy reported by Alkhodari et al. in 2021^19^.

The main difference between the proposed CNN-LSTM and the CNN-BiLSTM model proposed by Alkhodari et al.^19^ is that our proposed model uses a smaller number of parameters (28,277) compared to the number of parameters used by Alkhodari et al.^19^ since they use two LSTM layers instead of a single LSTM, they also have a larger input size and more convolution filters. In addition, the proposed CNN-LSTM system is tested both in the time and frequency domains while other systems only use the time domain or frequency domain. Moreover, other methods including Alkhodari et al.^19^ performed several pre-processing techniques like z-score normalization, smoothing, segmentation, and maximal overlap discrete wavelet transform (MODWT) while the proposed methodology performed downsampling only to decrease the number of samples to 8000 in the time domain and 1000 samples in the frequency domain for the whole signal without segmentation. In total, all of these parameters make the proposed CNN-LSTM system lighter compared to other models proposed in the literature.

Since the proposed methodology was built and trained using a CPU-based system, not a GPU-based system, and to demonstrate that it is a lightweight model. The time consumption of FFT computation, CNN-LSTM using time domain input, and CNN-LSTM using frequency input was calculated for all datasets and the results are displayed in Table 14. Rapid classification and FFT computation, combined with the high accuracy obtained using all datasets, and the small number of layers used are considered the main advantages of the suggested methodology. The result is a lightweight model that can be implemented using embedded systems.

**Table 14:** Time consumption of the proposed methodology.

Dataset	Process time (millisecond)		
	FFT computation	CNN-LSTM	FFT-CNN-LSTM
Multi classes dataset	1.0217	1.6283	1.9569
Augmented multi classes dataset	1.0423	1.8627	2.5631
Binary dataset	0.9468	1.6279	1.8964
Augmented binary dataset	1.0101	1.6752	2.1291
Average	1.005225	1.6985	2.136375

## Conclusions

Heart valvular irregularities are a major contributor to cardiovascular diseases (CVDs). This paper proposed an intelligent automatic heart diagnostic support system that uses phonocardiogram (PCG) signals. The model is hybrid and is comprised of a CNN module for feature extraction and an LSTM module for the classification of anomalies. For the multiclass problem using the open heart sounds dataset utilizing the time domain input, the end-to-end framework demonstrated state-of-the-art performance with 99.87% accuracy for augmented data and 98.5% accuracy for non-augmented data outperforming all prior efforts. The results also showed that augmenting the data slightly improved model performance by 1.37%. For the binary class problem using the PhysioNet/CinC 2016 challenge dataset, accuracy was 93.76%. On the other hand, utilizing the frequency domain input, the accuracy was 95.40% for non-augmented data and 99.73% for augmented data. The results also showed that augmenting the data improved model performance by 4.33%. For the binary class problem using the PhysioNet/CinC 2016 challenge dataset, accuracy was 90.65%. In the future, ECG signals can be used alongside PCG signals to design a multimodal system to improve accuracy. Moreover, this near perfection accuracy will be used to build a lightweight system that will help doctors performing clinical diagnostics discriminate all four irregularities early and quickly.

### Study limitations

This study has several advantages, including the potential use of cardiac PCG recordings to aid in the clinical decision-making of heart valve health. In addition to providing the highest levels of performance, the system was designed to be as simple as possible. The suggested model is easy to use, and it does not involve any modifications of the input signals. Despite the model's strong performance in categorizing heart valve disorders, it is critical to evaluate the suggested model using a wide variety of datasets that include more classes and records. While achieving a high level of discrimination using a simple deep neural network design, we may be able to improve the model's performance even further.

### Future study

In the future, we will focus on how to handle the sounds of more classes and extend the model to predict other heart diseases other than valvular ones. In addition, we will focus on implementing the suggested light model using embedded systems for the real-time prediction of diseases.

## Data Availability

The datasets generated and/or analyzed during the current study are available on GitHub (https://github.com/yaseen21khan/Classification-of-Heart-Sound-Signal-Using-Multiple-Features-/blob/master/README.md) and PhysioNet (https://archive.physionet.org/pn3/challenge/2016/) repositories.
